# A Case of Pulmonary Carcinoid Tumor with a Superimposed Aspergilloma Presenting As a Covert Ectopic Adrenocorticotropic Hormone Syndrome

**DOI:** 10.3389/fendo.2017.00123

**Published:** 2017-06-08

**Authors:** Kyoung Jin Kim, Ji Hee Yu, Nan Hee Kim, Young Hye Kim, Young Sik Kim, Ji A Seo

**Affiliations:** ^1^Division of Endocrinology, Department of Internal Medicine, Korea University College of Medicine, Seoul, South Korea; ^2^Department of Pathology, Gil Medical Center, Gachon University, Incheon, South Korea; ^3^Department of Pathology, Korea University College of Medicine, Seoul, South Korea

**Keywords:** Cushing syndrome, ectopic adrenocorticotropic hormone syndrome, pulmonary carcinoid tumor, aspergilloma, hypercortisolism

## Abstract

Ectopic adrenocorticotropic hormone (ACTH) syndrome is a challenging diagnosis only responsible for approximately 10% of Cushing syndrome cases. It has been associated with a variety of benign and malignant tumors including a carcinoid tumor accompanied by aspergilloma in our case that was significantly difficult to be detected. We report a patient over 70 years old with uncontrolled hypertension and hypokalemia presenting with generalized edema. Laboratory results revealed ACTH-dependent Cushing syndrome, but imaging studies did not show any discrete lesions secreting ACTH. The petrosal to peripheral ACTH gradient resulted in no evidence of pituitary adenoma. As the only lesion suspicious for ectopic ACTH secretion was a right lower round cystic lesion that did not appear to be a carcinoid tumor on computed tomography scan of the chest, the patient underwent video-assisted thoracic surgical resection to provide a definitive diagnosis. The final diagnosis was a small ectopic ACTH-secreting carcinoid tumor with unusual superimposed aspergilloma in the periphery of the lung. Postoperatively, the abnormal endocrine levels were normalized, and all of the clinical symptoms and signs were ameliorated. This is an informative case of ectopic ACTH syndrome (EAS) that was the cause of hypokalemia, hypertension, metabolic alkalosis, and hypercortisolism despite its poorly specific cushingoid morphology and uncommon imaging findings. Therefore, we recommend that clinicians investigate any possible lesion as a potential source of EAS.

## Introduction

The differential diagnosis and proper management of adrenocorticotropic hormone (ACTH)-dependent Cushing’s syndrome is one of the most intriguing challenges for endocrinologists (1). Most cases of ACTH-dependent Cushing syndrome are caused by corticotroph pituitary adenomas (90%), while ectopic ACTH production occurs in approximately 10% of cases (2). Ectopic ACTH syndrome (EAS) is defined as endogenous hypercortisolism caused by ACTH-secreting non-pituitary solid tumors, most of which originate in the chest cavity (3, 4). Accurately localizing the source of the ACTH-secreting lesion is crucial for resolving the condition through the removal of the primary ACTH-producing tumor (5, 6). However, the tumor might be very difficult to identify in instances of occult EAS because the deteriorate features of hypercortisolism can be an obstacle for further evaluation (7). For this reason, primary lesions of EAS have been undetected in 12–19% of tumors (8). Aspergilloma in carcinomatous cavities of lung or invasive pulmonary aspergillosis in immunocompromised patients with lung cancer is commonly found (9). However, non-cavitary lung tumors complicated by aspergillosis in immunocompetent patients are rare. Regarding pulmonary carcinoids, there are a few cases of endobronchial carcinoids with coexisting aspergillosis (10).

Here, we report a very rare case of EAS stemming from a peripheral lung lesion that had remained unchanged for 3 years and that was finally diagnosed as a small pulmonary carcinoid tumor adjacent to aspergilloma.

## Case Report

A patient over 70 years old was admitted with bilateral pedal edema and generalized weakness that had become apparent 1 month earlier. There were no underlying chronic lung disease and no history of smoking. The patient also complained of unintentional weight gain (5 kg) with proximal muscle weakness. Upon physical examination, the blood pressure was 170/90 mmHg although a combination of four antihypertensives had been taken. The patient presented with a puffy face, central obesity without purplish striae, and thin skin with pitting edema, none of which are specific to cushingoid symptoms.

**Figure 1:**
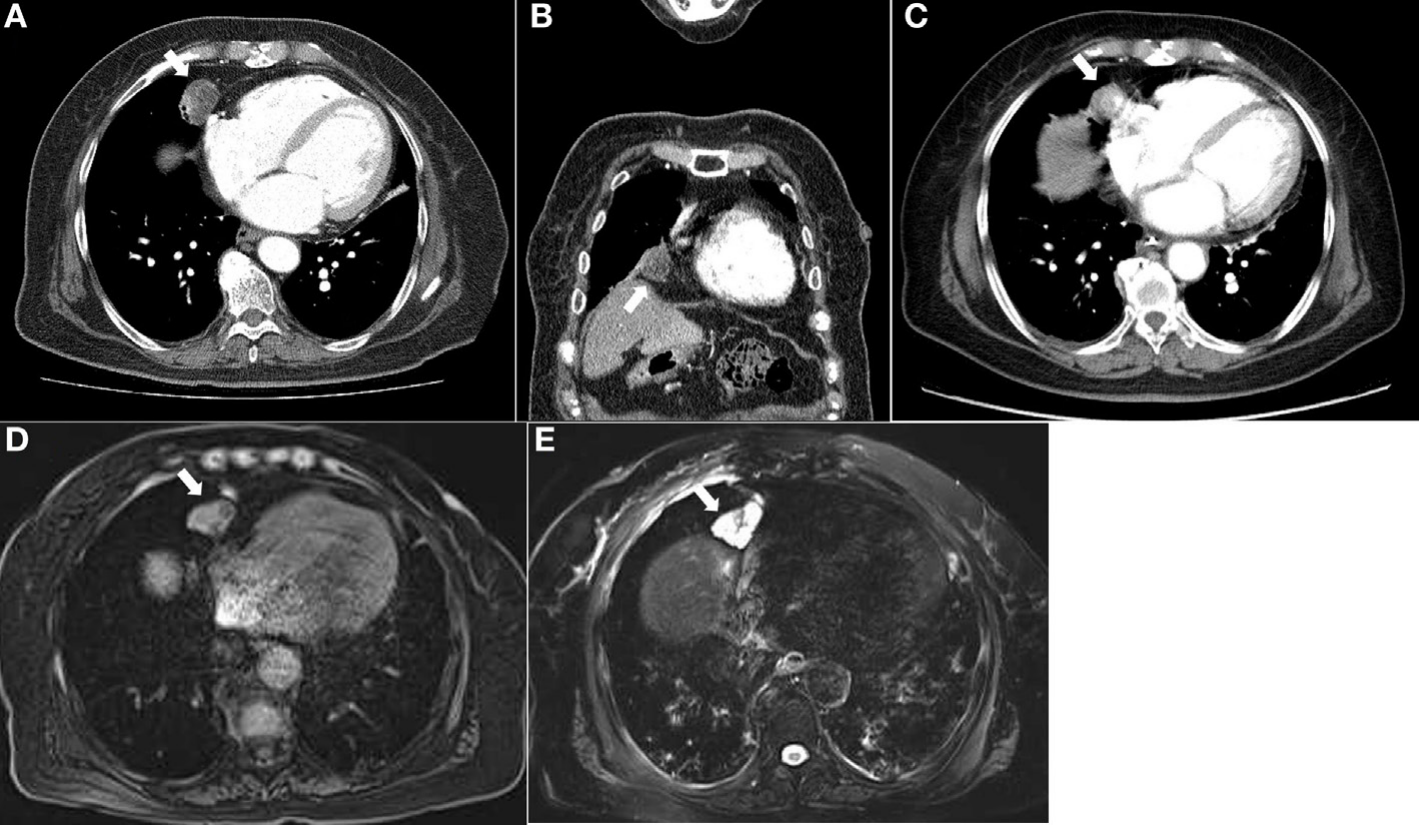
Computed tomography of the chest demonstrated a round-shaped, poorly enhancing mass with cystic portions measuring 2.7 cm at the right anterior cardiophrenic angle in the axial **(A)** and coronal **(B)** images. This lesion was observed on an incidenta abdominal CT scan taken 3 years ago. **(C)** Magnetic resonance imaging of the chest showed a lobulating mass at the right anterior cardiophrenic angle, with intermediate signal intensity on the T1-weighted image **(D)** and high signal intensity on the T2-weighted image **(E)**.

The laboratory results are summarized in Table 1. The potassium level was extremely low, and the trans-tubular potassium gradient was 5. The patient was also hyperglycemic. The blood gas analysis was indicative of metabolic alkalosis with a pH of 7.628 and HCO_3_ level of 46.1 mmol/L. We investigated the status of mineralocorticoid excess. Plasma renin activity and aldosterone were within normal ranges (Table 1). Urine free-cortisol excretion was markedly increased with elevated morning serum cortisol, which was not suppressed after a 1 mg overnight dexamethasone suppression test (DMST) (Table 1). The basal ACTH was elevated, which led us to expect ACTH-dependent Cushing syndrome. To differentiate Cushing’s disease from ectopic ACTH secretion, pituitary magnetic resonance imaging (MRI) was performed but revealed no lesion. Serum cortisol was not suppressed by an 8 mg high-dose DMST, implying EAS (Table 1). To localize the source of the ectopic ACTH, radiographic investigations were performed. Contrast-enhanced computed tomography (CT) of the thorax showed a round-shaped mass with cystic components measuring approximately 27 mm × 21 mm at the right anterior cardiophrenic angle, with a partial contrast enhancement (Figures 1A,B). The mass was unchanged over 3 years based on an incidental abdominal CT scan owing to acute cholecystitis (Figure 1C). On the CT of the abdomen, both adrenal glands were diffusely thickened without any evidence of a mass. As there was no discrete lesion of an ectopic ACTH-secreting tumor, we implemented a 2-day standard high-dose DMST (2 mg every 6 h for a total of eight doses), in which 24 h urine free cortisol decreased 91% from baseline (Table 1). No definite source of EAS could be found even after all of the examinations. However, the patient refused further intervention and was discharged with high-dose potassium supplements, angiotensin receptor blockers, and aldosterone antagonists.

**Table 1:** Results of laboratory tests.

Variable	Result	Reference range
Hemoglobin, g/dL	11.3	12.3–15.3 (women)
Total leukocytes, /μL (neutrophil %)	10,800 (83.7%)	4,000–11,000
Serum potassium, mmol/L	2.0	3.4–5.3
Serum glucose (random), mg/dL	396	<140
HbA1c, %	7.0	<6.0
Thyroid-stimulating hormone, μIU/mL	0.2	0.17–4.05
Free thyroxine, ng/dL	1.19	0.79–1.86
Upright, plasma aldosterone, ng/dL	2.1	6–25
Upright, plasma renin activity, ng/mL/h	0.71	0.29–3.7
Morning serum cortisol, μg/dL	43.0	9.4–26.0
Morning plasma ACTH, pg/mL	77.3	<60
Midnight serum cortisol, μg/dL	43.2	<5–7.5
24 h urine free cortisol, μg/day	5,380.6	35–150
1 mg overnight DMST cortisol, μg/dL	43.0	<1.8
^a^8 mg overnight DMST cortisol, μg/dL	30.0	Cortisol >50% suppression after DMST indicating Cushing’s disease
^b^High-dose DMST cortisol, μg/dL	29.7	
^b^High-dose DMST 24 h urine free cortisol, μg/day	464.0	

*ACTH, adrenocorticotropic hormone; DMST, dexamethasone suppression test*.

*^a^About 8 mg dexamethasone was administered orally between 11:00 p.m. and midnight, and a single blood sample was drawn at 8:00 a.m. the next day*.

*^b^About 2 mg dexamethasone was administered orally every 6 h for 2 days, with 16 mg total administered during the test at 8:00 a.m., 2:00 p.m., 8:00 p.m., and 2:00 a.m., and a blood specimen was drawn 6 h after the last dose. The urine collections were continued during the 2 days that dexamethasone was administered*.

**Figure 2:**
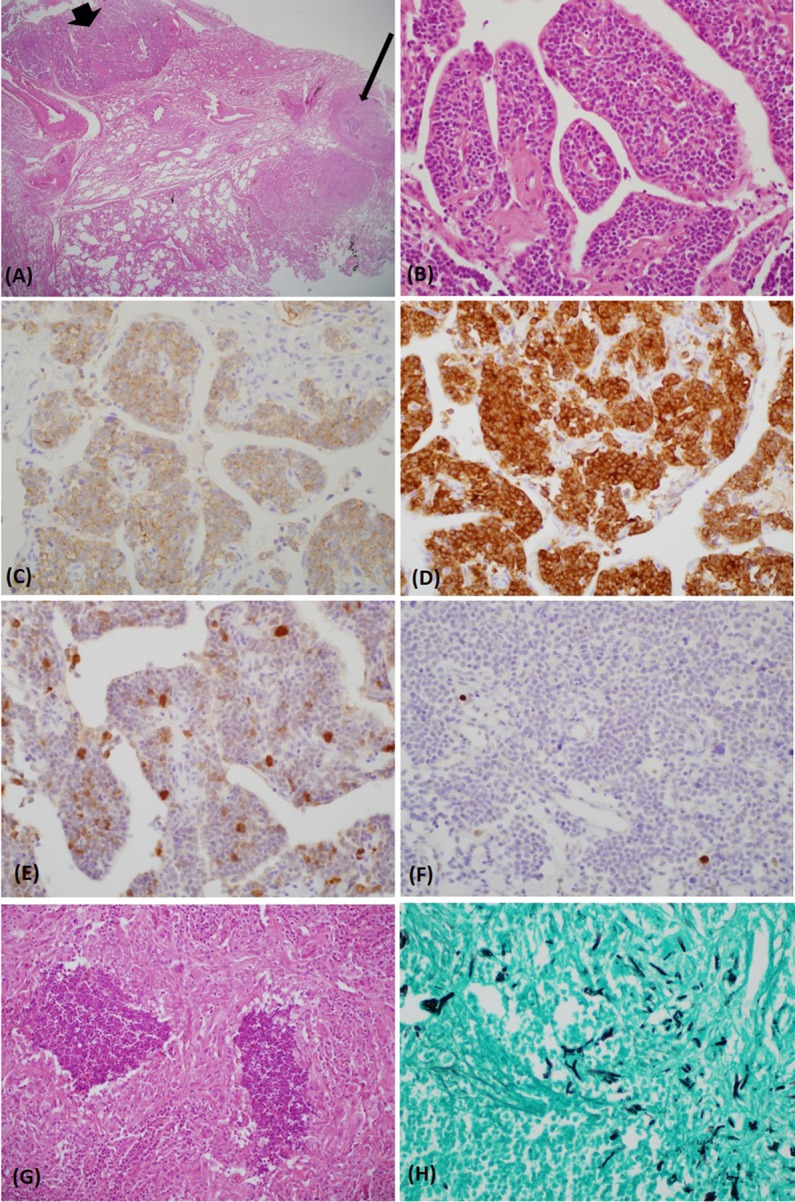
The carcinoid tumor (bold arrow) and necrotizing granuloma (thin arrow) were in close proximity [hematoxylin and eosin (H&E) stain, ×12.5] **(A)**. The tumor consisted of uniform polygonal cells with a salt–pepper chromatin pattern (H&E stain, ×400) **(B)**. Upon immunohistochemical staining, the tumor was immunoreactive for CD 56 **(C)**, synaptophysin **(D)**, and adrenocorticotropic hormone **(E)**. The Ki-67 index was very low (<2%) **(F)**. A lesion abutting the carcinoid tumor exhibited chronic granulomatous inflammation with necrosis (H&E stain, ×400) **(G)**. Gomori methenamine-silver stain revealed acutely branching and septated fungal hyphae within the necrotic tissue (×400) **(H)**.

One month after discharge, the patient was readmitted because of progressive generalized edema with sustained hypokalemia. The patient underwent bilateral inferior petrosal sinus sampling, which revealed no petrosal to peripheral ACTH gradient (Table 2). On chest MRI, a lobulating nodule was found corresponding to the location indicated by CT (Figures 1D,E). The patient declined to undergo [^18^F]-fluorodeoxyglucose positron emission tomography (FDG-PET) or nuclear scans for financial reasons. Since no suspicious foci other than the chest lesion were found, a wedge resection under video-assisted thoracoscopic surgery was performed. Macroscopically, the tumor was a relatively well-demarcated and ivory-colored nodule measuring 16 mm × 15 mm. It was adjacent to another grayish yellow mass measuring 33 mm × 22 mm. Microscopically, the two lesions were in very close proximity, presenting as one lesion radiographically (Figure 2A). The tumor consisted of uniform polygonal cells with finely granular chromatin and low mitotic activity, which histologically favored a typical carcinoid lesion (Figure 2B). It originated from the parenchyma adjacent to the visceral pleura. By immunohistochemistry, the tumor cells were positive for neuroendocrine markers (CD 56 and synaptophysin) and ACTH, with a low proliferation rate (<2%) on Ki-67 staining. This confirmed the final diagnosis of a carcinoid tumor with ACTH expression (Figures 2C–F). The adjacent grayish yellow mass exhibited chronic necrotizing granulomatous inflammation with *Aspergillus*, highlighted by Gomori methenamine-silver staining (Figures 2G,H).

**Table 2:** Results of inferior petrosal sinus sampling using CRH as a stimulator.

	Peripheral ACTH, pg/mL	Petrosal ACTH (petrosal/peripheral ACTH ratio)	
		Right, pg/mL	Left, pg/mL
Basal	165.1	179.4 (**1.09**)	181.7 (**1.10**)
Peak	153.9	215.6 (**1.40**)	171.6 (**1.11**)

*ACTH, adrenocorticotropic hormone; CRH, corticotropin-releasing hormone*.

Following resection of the lung lesion, plasma ACTH and 24 h urine free cortisol returned to normal. The potassium level was normalized without supplements, and the edema subsided. Over more than 1 year of follow-up appointments, the patient has been free from hypercortisolism. This case was carried out in accordance with the recommendations of the Institutional Review Board from the Korea University Medical Center. The written informed consent was obtained from the patient.

## Discussion

Aspergillosis coexisting with pulmonary carcinoid tumors has rarely been reported. We found only seven cases of pulmonary carcinoids associated with aspergillosis in a review of the literature (9). In all reported cases, the tumors were localized centrally in the bronchus, while our patient exhibited a peripheral lung lesion. To the best of our knowledge, this is the first report of an ectopic ACTH-secreting pulmonary carcinoid tumor masked by aspergilloma that was not located in the bronchus.

Regarding the timing of the identification of the source of ACTH, EAS can be classified into three subgroups: overt, covert, and occult (7, 11). EAS is classified as overt when the tumor source is detected promptly after initial evaluation and covert when the tumor source is identified after prolonged follow-up evaluation. A diagnosis of occult EAS is reserved for patients whose tumor source cannot be found even after a meticulous diagnostic workup (7, 11). When our patient was initially admitted owing to hypercortisolism, the round mass at the right anterior cardiophrenic angle was scarcely suspected as the source of ACTH. The radiologists initially suggested a diagnosis of thymolipoma, thymoma, inflamed pericardial cyst, or thymic cyst because the lesion was not typical of a carcinoid tumor. According to Meisinger et al., peripheral pulmonary carcinoid tumors have lobulated or high attenuated features on contrast-enhanced CT or appear with subsegmental airway obstruction, none of which were visible in our case (12). Moreover, the patient had been asymptomatic for over 3 years, and the mass was unchanged. The lesion was heterogenous on chest CT and MRI, which indicated the possibility of a mix of two different lesions. However, the structures with contrast enhancement on CT and high signal intensity on MRI were not correlated. Therefore, it was very difficult to define the lesion as two separate lesions by imaging, even upon retrospective review. This is a case of covert EAS, because the source of ACTH could not be easily suspected through imaging, and the diagnosis was delayed until an explorative surgical procedure was performed.

A variety of benign and malignant tumors have been associated with EAS (1, 6). The most common site for EAS is the lung, caused by either a small cell lung tumor or bronchial carcinoid tumor (11). In this case, the tumor secreting ACTH was proven a pulmonary carcinoid tumor attached to a chronic pulmonary aspergilloma located in the periphery of the lung. Non-carcinoid tumors associated with aspergillosis frequently have a cavity owing to central necrosis, a result of postobstructive cyst formation by an occluding bronchial lumen, or scar carcinoma in a preexisting cavity. Those patients usually have a history of long-term smoking (10). On the contrary, when aspergillosis coexists with lung carcinoid tumors, most tend to be localized centrally in either a main or lobar bronchus presenting as an obstructive endobronchial mass without cavitary formation (10). In those cases, the patients frequently had risk factors for chronic lung disease (10). However, our patient had no risk factors for chronic lung disease and no bronchial obstructive symptoms because the tumor was not an endobronchial lesion. It was unclear whether the aspergilloma or carcinoid tumor occurred first in this case. When carcinoids and *Aspergillus* are combined, they can be either coincidental or secondary to mutual risk factors (10). Nilsson et al. suggested two hypotheses to explain the order of occurrence. First, *Aspergillus* releases digestive enzymes, such as aflatoxin, that contain tumorigenic properties and may contribute to cavitation. Second, solid tumors locally impair the immune defense and diminish airway clearance, which can create an environment for increased fungal growth, such as aspergilloma (10). Considering the low mitotic count of the carcinoid tumor, non-invasive nature of the aspergillosis, and at least a 3 years of asymptomatic period after initial detection of the pulmonary lesion, we believe that the aspergilloma developed first and then the carcinoid developed, or *vice versa*.

Invasive fungal infections such as aspergillosis and cryptococcosis occur commonly in EAS when the hypercortisolism is sufficiently severe to impair cell-mediated immunity (13). However, the aspergillosis in our patient was non-invasive and indolent, which had not been reported previously. The optimal strategy for antifungal treatment of aspergillosis coexisting with lung carcinoma has yet to be established (9). Nevertheless, we concluded that antifungal therapy was unnecessary, given that our patient was comparatively immunocompetent after successful removal of the tumor.

All diagnostic efforts should focus on the identification of the source of ACTH production because the risk of morbidity and mortality increases continuously without curative resection of the source (14). Because most tumors causing EAS are intrathoracic, imaging modalities on the chest should be performed carefully (15). In the case of EAS, the anatomical imaging modalities are CT and MRI, and functional imaging modalities are [^111^In]-diethylenetriaminepentaacetate-d-Phe-pentetreotide (octreoscan; OCT) and [^18^F] FDG-PET. The combination of two anatomic methods, CT and MRI, increases detection, with a per-patient sensitivity of 83% and a positive predictive value per lesion of 80% (15). Functional imaging can be useful when CT and MRI results are questionable, because functional imaging modalities can highlight specific properties of tumor cells, not just anatomical characteristics (15). The ability of OCT to identify tumors depends on multiple factors, including lesion size, location, and type and degree of somatostatin receptor expression (16). OCT can detect a true ectopic ACTH lesion when anatomical imaging modalities are insufficient (15, 16). Meanwhile, FDG-PET has been reported to be variably helpful, but no data support its increased effectiveness compared to CT or MRI scanning considering the cost-effectiveness, especially when the tumors have low metabolic activity (15–17). Unfortunately, our patient refused to undergo functional imaging for financial reasons. Small peripheral bronchopulmonary carcinoids can be easily missed on thorough chest evaluations (18). Pulmonary carcinoid tumors presenting as solitary pulmonary nodules should be considered when they appear as lobulated nodules of high attenuation on contrast-enhanced CT, are calcified, or are associated with distal bronchial lesions (12). In this case, the carcinoid tumor could not be distinguished from benign nodules by imaging, with no typical features of a usual peripheral pulmonary carcinoid tumor. Only partial contrast enhancement by CT provided a subtle clue to suggest a carcinoid tumor, which impeded the diagnosis and treatment.

Concerning the management of EAS, surgical excision is the first treatment of choice once a single source of the ectopic tumor is successfully localized. Approximately 83% of EAS patients with bronchial carcinoid tumors recover after radical excision (4, 11). If curative resection is not successful, medications including adrenal enzyme inhibitors and glucocorticoid antagonists should be considered to control the deleterious consequences of hypercortisolemia (11). Furthermore, a bilateral adrenalectomy with steroid replacement is an alternative treatment when the primary tumor cannot be found and medicinal controls fail (4). Fortunately, our patient endured hypercortisolism with the only necessary treatment being successful surgical resection of the source. The combined efforts of endocrinologists and expert radiologists will enhance the identification of ectopic ACTH lesions and allow surgeons to attempt curative resection for the complete remission of hypercortisolism (7). This highlights the importance of multidisciplinary approaches to treat EAS.

## Conclusion

We report a very rare case of covert EAS from a pulmonary carcinoid tumor with coexisting aspergilloma that was not in an endobronchial location. Fortunately, the resection of the lesion managed to resolve the hypercortisolemia. Although the diagnosis can be challenging, especially in the evaluation of patients with a hidden primary tumor, we must adopt a multidisciplinary approach to localize and remove the source of hypercortisolism to prevent serious complications, considering the possibility of a masked lesion as the etiology.

## Ethics Statement

This case report was carried out in accordance with the recommendations of the Institutional Review Board from the Korea University Medical Center.

## Author Contributions

KJK is the first author for this case report. JAS is the corresponding author supervising this work. JHY, NHK, YSK, and YHK are coauthors performing analysis on all data interpretation and manuscript evaluation.

## Conflict of Interest Statement

The authors declare that the research was conducted in the absence of any commercial or financial relationships that could be construed as a potential conflict of interest.

## Abbreviations

ACTH, adrenocorticotropic hormone; EAS, ectopic ACTH syndrome; DMST, dexamethasone suppression test; MRI, magnetic resonance imaging; CT, computed tomography; Octreoscan, [^111^In]-diethylenetriaminepentaacetate-d-Phe-pentetreotide.
